# Correlation between Motor Cortex Excitability Changes and Cognitive Impairment in Vascular Depression: Pathophysiological Insights from a Longitudinal TMS Study

**DOI:** 10.1155/2016/8154969

**Published:** 2016-07-20

**Authors:** Manuela Pennisi, Giuseppe Lanza, Mariagiovanna Cantone, Riccardo Ricceri, Concetto Spampinato, Giovanni Pennisi, Vincenzo Di Lazzaro, Rita Bella

**Affiliations:** ^1^Spinal Unit, Emergency Hospital “Cannizzaro”, 95126 Catania, Italy; ^2^Department of Neurology I.C., “Oasi” Institute for Research on Mental Retardation and Brain Aging (I.R.C.C.S.), 94018 Troina, Italy; ^3^Department of Medical and Surgical Sciences and Advanced Technologies, Section of Neurosciences, University of Catania, 95125 Catania, Italy; ^4^Department of Electrical, Electronics and Informatics Engineering, University of Catania, 95125 Catania, Italy; ^5^Department of Surgery and Medical-Surgical Specialties, University of Catania, 95125 Catania, Italy; ^6^Department of Medicine, Unit of Neurology, Neurophysiology, Neurobiology, Campus Bio-Medico University of Rome, 00128 Rome, Italy

## Abstract

*Background*. Transcranial magnetic stimulation (TMS) highlighted functional changes in dementia, whereas there are few data in patients with vascular cognitive impairment-no dementia (VCI-ND). Similarly, little is known about the neurophysiological impact of vascular depression (VD) on deterioration of cognitive functions. We test whether depression might affect not only cognition but also specific cortical circuits in subcortical vascular disease.* Methods*. Sixteen VCI-ND and 11 VD patients, age-matched with 15 controls, underwent a clinical-cognitive, neuroimaging, and TMS assessment. After approximately two years, all participants were prospectively reevaluated.* Results*. At baseline, a significant more pronounced intracortical facilitation (ICF) was found in VCI-ND patients. Reevaluation revealed an increase of the global excitability in both VCI-ND and VD subjects. At follow-up, the ICF of VCI-ND becomes similar to the other groups. Only VD patients showed cognitive deterioration.* Conclusions*. Unlike VD, the hyperfacilitation found at baseline in VCI-ND patients suggests enhanced glutamatergic neurotransmission that might contribute to the preservation of cognitive functioning. The hyperexcitability observed at follow-up in both groups of patients also indicates functional changes in glutamatergic neurotransmission. The mechanisms enhancing the risk of dementia in VD might be related either to subcortical vascular lesions or to the lack of compensatory functional cortical changes.

## 1. Introduction

Among the factors that contribute to cognitive decline in older adults, there is now agreement that depression of the late life is one of the recognized clinical risk factors for dementia [1–3]. In a recent clinical-pathological study, the presence of depression prior to the onset of dementia was found to be more common and more drug-resistant in patients with vascular dementia (VaD) than in those with Alzheimer's disease (AD) [4]. A close relationship between depression, cognitive impairment, and cerebrovascular pathology has been also reported in vascular depression (VD), based on the evidence that white matter lesions (WMLs) are more common and more severe in individuals with late-onset depression than in healthy subjects or in patients with early-onset depression [5]. In particular, it has been hypothesized that the effects of the ischemic damage of the frontal cortical-subcortical circuits implicated in mood-affect regulation and cognition may result in depressive symptoms and executive dysfunction [6, 7]. Subcortical vascular lesions might disrupt critical pathways acutely, such as the basal ganglia in the poststroke depression [8], or through progressive axonal damage on glutamatergic, GABAergic, or catecholamine neuronal circuits along the frontal-striatal pathways [9]. Moreover, several studies suggest that VD patients have a poor response to antidepressant treatment [10] and are at higher risk of mortality and cognitive impairment compared to nondepressed individuals [11, 12]. However, the functional mechanisms that enhance the risk of dementia in older adults with depression are still not entirely understood.

More recently, transcranial magnetic stimulation (TMS) has provided the opportunity for the noninvasive functional assessment of glutamatergic, cholinergic, and GABAergic circuits of the human motor cortex [13]. Several studies have used this technique to assess inhibitory and excitatory interactions within cortical regions in several neuropsychiatric disorders [14], such as cognitive decline and depressive disorders. In some studies, functional evaluation has been performed longitudinally in patients with an overt dementia, and it has been shown that there is a progressive enhancement in excitatory cortical neurotransmission both in AD and in subcortical ischemic VaD patients [15–17]. Using TMS techniques, another form of functional rearrangement of the central motor circuits, with a clear medial and frontal shift of the motor areas, has been reported both in AD and in subcortical ischemic VaD [18, 19]. Taken together, these findings suggest the existence of functional changes in central motor circuits which are common to degenerative and vascular cognitive disorders. The significance of these changes is still unknown, although they might provide protection against the decline in motor programming and execution which could be induced by disease progression [20]. At present, little is known about the functional changes that take place in patients with vascular cognitive impairment-no dementia (VCI-ND) at risk for future VaD and the impact of late-onset depression on any plastic change which could contribute to the preservation of cognitive functions.

To better characterize the possible role of depression in cognitive decline of patients with vascular damage, we investigated the relationship between the progression of the neurophysiological changes and cognitive impairment in patients with VCI-ND with those obtained in a group of VD patients and controls. Our hypothesis was that the presence of late-onset depression might affect not only cognition but also the functioning of specific cortical circuits which can be explored by TMS.

## 2. Materials and Methods

### 2.1. Participants

Sixteen VD (68.1 ± 8.6 years) and eleven VCI-ND patients (70.0 ± 7.0 years) were consecutively recruited from the Cerebrovascular Disease Center of the University of Catania (Italy) and compared with fifteen age-matched controls (63.8 ± 7.2 years). Participants were included as VCI-ND when they meet the imaging criteria for subcortical vascular disease with predominant WMLs [21]. They also did not satisfy the criteria for dementia according to the Diagnostic and Statistical Manual for Mental Disorders-Forth Edition-Text Revised (DSM-IV-TR), although they were required to show deterioration in at least one cognitive domain but normal functional status in their activities of daily living [22]. Exclusion criteria were as follows: major neurological disorders, such as dementia, stroke, Parkinson's disease, multiple sclerosis, head trauma, and epilepsy; psychiatric illness, including depressive disorders; acute medical illness or organ failure (i.e., heart failure, liver cirrhosis, kidney failure, respiratory failure, severe metabolic imbalance, and diffuse neoplasm); alcohol or drug abuse; score at mini mental state examination (MMSE) [23] <24; exposure to drugs able to affect cortical excitability, such as benzodiazepines, zolpidem, antipsychotics, mood stabilizers, and antiepileptic drugs; and any condition precluding Magnetic Resonance Imaging (MRI) or TMS execution. None of the VCI-ND participants was on antidepressant, other psychotropic drugs, or cholinesterase inhibitors medications.

VD participants were required to fulfill the DSM-IV-TR diagnostic criteria for unipolar major depressive disorder and MRI criteria for subcortical vascular disease with predominant WMLs [21]. Before the enrolment, 3 VD patients were on tricyclic antidepressant, whereas 6 and 7 of them were treated with Selective Serotonin Reuptake Inhibitors and Serotonin Noradrenaline Reuptake Inhibitors, respectively. A pharmacological wash-out was performed two weeks before any TMS procedure, as recommended [24]. Patients with a history of major psychiatric illness (except for personality disorders and anxiety, if secondary to depression), major neurological disorders (see above), history of epilepsy, acute medical illness or organ failure, mood or cognitive disorder due to endocrinopathies, alcohol or drug abuse, intake of drugs causing depressive symptoms (i.e., steroids, beta-blockers, and clonidine) or modulating cortical excitability (see above), MMSE < 24, and contraindication to MRI or TMS were excluded.

Conventional electroencephalography was performed prior to the enrolment to rule out predisposition to seizures. The study was approved by the local Ethics Committee based at the “Policlinico-Vittorio Emanuele” University Hospital of Catania (Italy), and written informed consent was obtained from all participants prior to the participation, in accordance with the Declaration of Helsinki. All assessments were performed in a controlled laboratory environment.

### 2.2. Assessment

All subjects underwent clinical assessment, including age, gender, education, handedness, presence of cardiovascular risk factors (hypertension, diabetes, hypercholesterolemia, coronaropathy, atrial fibrillation, and smoking habit), and general and neurological examinations. Patients and controls were treated for their vascular risk factors with antiplatelet or anticoagulant medications (aspirin, clopidogrel, and warfarin), antihypertensive drugs (angiotensin converting enzyme inhibitors, angiotensin II receptor antagonist, diuretics, and calcium channel blockers), cholesterol lowering medications (statins), and oral antidiabetic drugs or insulin.

None of the patients had focal neurological deficit. The three groups were similar in terms of educational level and vascular risk factors profile; VD participants exhibited a more frequent family and personal history of depression. The neuropsychological battery of tests assessed overall cognitive impairment (MMSE) [23], frontal lobe abilities (frontal assessment battery, FAB) [25], and the interference task Stroop color-word test (total time, Stroop T, and number of errors, Stroop E) [26]. The presence of depressive symptoms and apathy was quantified by means of the 17-item Hamilton Depression Rating Scale (HDRS) [27] and the Apathy Scale (AS) [28], respectively. Functional status was defined by using the Activity of Daily Living (ADL) and the Instrumental Activity of Daily Living (IADL) scores.

Brain MRI was acquired from all participants with a 1.5 Tesla General Electric system. The imaging protocol consisted of T1-, T2-, and proton density-weighted and fluid-attenuated inversion recovery (FLAIR) scans; slice thickness was 5 mm with 0.5 mm slice gap. In all subjects, the severity of WMLs was graded according to the Fazekas visual scale [29]: 0 = absence; 1 = punctuate foci; 2 = beginning confluence of foci; and 3 = large confluent areas. Accordingly, WML severity was graded as mild in 8 VD patients (grade 1), moderate in 4 patients (grade 2), and severe in 4 patients (grade 3); 6 VCI-ND patients were rated as grade 1, 4 patients were rated as grade 2, and 1 patient was rated as grade 3; brain MRI was normal in all controls (grade 0).

### 2.3. Transcranial Magnetic Stimulation

TMS was performed using a high-power Magstim 200 mono pulse magnetic stimulator (Magstim Co., Whitland, Dyfed, UK). A 70 mm figure-of-eight coil was held over the motor cortex at the optimum scalp position to elicit the motor evoked potentials (MEPs) in the contralateral first dorsal interosseous (FDI) muscle of the dominant hand, according to the Edinburgh Handedness Inventory [30]. The flat surface of the coil was positioned tangentially on the scalp over the primary motor cortex. Electromyographic (EMG) activity was recorded from a silver/silver chloride surface active electrode placed over the motor point of the target muscle, with the reference electrode placed distally at the metacarpal-phalangeal joint of the index finger. Motor responses were amplified and filtered (bandwidth: 3–3000 Hz) using a 2-channel Medelec Synergy (Oxford Instruments Medical, Inc., UK) system with an amplification factor of the screen of 100 *μ*V per division unit for the measurement of resting motor threshold (rMT) and 1 mV per division unit during the MEP recording. The temporal resolution of the screen was 5 ms per division unit in such a way that the TMS artefact, the beginning, and the end of the MEP were always visible [13, 31].

For the motor nerve conduction study (M and F waves from the FDI muscle), a bipolar nerve stimulation electrode with 6 mm diameter felt pads and an interelectrode separation of 25 mm was used. M and F waves were elicited by giving supramaximal electrical stimulation (constant current square wave pulse of 0.2 ms) to the ulnar nerve at wrist. Three reproducible artefact-free M responses and ten F waves were recorded for each of the subjects. While FDI was relaxed, the peak-to-peak amplitude of M and F waves was determined. We identified the F waves according to the criteria reported by the International Federation of Clinical Neurophysiology (IFCN) as responses that are variable in their latency, amplitude, and configuration but that occur grouped with a consistent range of latency. The F wave with the shortest latency, providing a measure of conduction in the fastest motor axons, was considered [13, 31].

Measures of motor cortex excitability included resting motor threshold (rMT), cortical silent period (CSP), MEPs, and central motor conduction time (CMCT) from both hemispheres. Resting MT was defined, according to the IFCN Committee recommendation [13], as the lowest stimulus intensity able to elicit MEPs of an amplitude >50 *μ*V in at least 5 of 10 trials, with the muscle at rest. It is a global measure of cortical excitability reflecting the excitability of cortical-spinal neurons and interneurons projecting onto these neurons in the motor cortex and of spinal motor neurons, neuromuscular junctions, and muscle [13]. The CSP was determined with an approximately 50% of maximum tonic voluntary contraction of the FDI muscles, induced by contralateral TMS pulses delivered at 130% of the rMT. During the CSP recordings, the subjects maintained the isometric tonic contraction by abducting the index finger against a strain gauge. The mean CSP duration based on trial-by-trial measurements of 10 rectified traces was calculated. Following the IFCN guidelines [13, 31], in a single trial, the CSP was measured as the time elapsing from the onset of the MEP until the recurrence of voluntary tonic EMG activity. If voluntary EMG activity did not recover abruptly but gradually made the identification of the end of the CSP difficult, the following criteria on a single trial basis were used: when the EMG activity reached or exceeded the pre-TMS baseline level and lasts for at least 50 ms, reoccurring EMG activity marked the end of the CSP. As known, the CSP is mainly mediated by the activity of GABAergic intracortical neurons [13, 31]. CMCT reflects the integrity of the cortical-spinal tract, from the upper to the lower motor neurons. It was calculated by subtracting the conduction time in peripheral nerves from MEP latency obtained during moderate active muscle contraction (10–20% of maximum background force), at a stimulus intensity set at 130% of the rMT [13]. By using the F wave latency, CMCT (ms) was estimated as T − (F + M − 1)/2 [T is onset latency of MEP elicited by TMS; F is onset latency of F wave by electrical ulnar nerve stimulation; M is onset latency of M wave by electrical ulnar nerve stimulation] [31]. Moreover, in order to assess spinal motor excitability, the mean amplitude of the F wave was measured in the target muscle [32, 33].

Intracortical circuits were studied bilaterally using the conditioning test paradigm described by Kujirai and coworkers [34] through a BiStim module (Magstim Co., Whitland, Dyfed, UK) connected to a Cambridge Electronic Design (CED) Micro 1401 Interface (Cambridge, UK). The procedure consisted of applying two magnetic stimuli in rapid succession through two magnetic stimulators connected to each other. The conditioning stimulus was applied at 80% of the subject's rMT, and the intensity of the test stimulus was set at 130% of the rMT. The interstimulus intervals (ISIs) tested were 1, 3, 5, 7, 10, and 15 ms; ten trials for each ISI were recorded randomly. The responses were expressed as the ratio of the MEP amplitude produced by paired stimulation to that produced by test stimulation alone. Short-latency intracortical inhibition (SICI) was obtained at short ISIs in which the conditioning stimulus determines an inhibition with respect to the test stimulus; it is attributed to an activation of inhibitory neuronal system transmission [35, 36]. Intracortical facilitation (ICF) was studied at longer ISIs in which the conditioning stimulus determines an enhanced response with respect to the test stimulus; it is modulated by multiple neurotransmission pathways, although mainly through excitatory glutamatergic neurons [37, 38].

All TMS measurements were conducted, while subjects were seated in a comfortable chair with continuous EMG monitoring to ensure either a constant level of muscular activity during tonic contraction or complete relaxation at rest. Data were collected on a computer and stored for offline analysis. Hardware setting, data collection, and offline processing were performed by using an* ad hoc* tool which is detailed in the article by Giordano and coworkers [39].

### 2.4. Follow-Up

All participants were reevaluated after a median period of approximately two years (VD: 24.1 ± 2.1 months; VCI: 23.9 ± 1.8 months; controls: 23.2 ± 1.7 months; *p* = 0.15), with the same assessment performed at the entry of the study, including clinical-demographic evaluation, neuropsychological tests, and single- and paired-pulse TMS. Brain MRI was repeated in all patients, showing a progression of the vascular burden, from grade 1 to grade 2, in one VCI-ND patient and in two VD patients. Of the original cohort, one VCI-ND patient and one VD patient were no more eligible to TMS due to a permanent pacemaker implantation and the poor medical condition, respectively; nevertheless, these patients were reassessed for the cognitive profile.

### 2.5. Statistical Analysis

The nonparametric Kruskal-Wallis ANOVA test was used for the comparison of clinical, neuropsychological, and neurophysiological parameters of patients and controls at baseline (time *t*_0_) and at follow-up (time *t*_1_) and their differences (assessed as *t*_1_ − *t*_0_). The Mann-Whitney test was employed as a* post hoc* analysis for the pairwise comparison. The Wilcoxon test for paired data sets was used for the comparison of clinical, neuropsychological, and neurophysiological variables at times *t*_1_ and *t*_0_ for each group patient. Nonparametric statistics analysis was required given the categorical nature of the neuropsychological testing results and the nonuniform distribution of the results of the TMS studies. Correlations between neuropsychological and TMS variables were evaluated by means of Spearman's correlation coefficient. A *p* value lower than 0.05 was considered as statistically significant. To account for multiple comparison, Bonferroni correction and Benjamini-Hochberg procedure were employed.

## 3. Results and Discussion

### 3.1. Baseline

**Figure 1:**
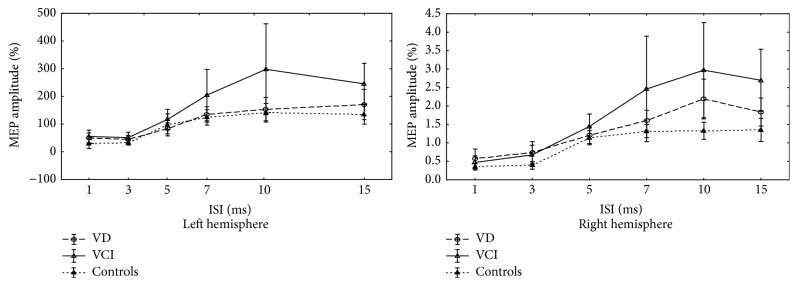
The mean time course of intracortical excitability in the patients and controls at baseline. MEP, motor evoked potential; ISI, interstimulus interval; VD, patients with vascular depression; VCI, patients with vascular cognitive impairment-no dementia.

Neuropsychological characteristics of all participants at the entry of the study are summarized in Table 1. As shown in Table 2, no statistically significant differences between patients and controls were found at baseline for single-pulse TMS parameters. The mean time course of intracortical excitability of all subjects at time *t*_0_ is shown in Figure 1. At baseline, there was a significantly more pronounced ICF in VCI-ND patients than in controls and in VD patients. In detail, conditioned MEP amplitude from both hemispheres at ISI of 10 ms (*left hemisphere*: VD, 1.5 ± 0.9; VCI-ND, 3.0 ± 2.7; controls, 1.4 ± 0.6; *p* = 0.0009;* right hemisphere*: VD, 1.6 ± 0.6; VCI-ND, 2.5 ± 2.4; controls, 1.3 ± 0.3; *p* = 0.0092) and at ISI of 15 ms (*left hemisphere*: VD, 1.7 ± 1.1; VCI-ND, 2.5 ± 1.2; controls, 1.3 ± 0.7; *p* = 0.0021;* right hemisphere*: VD, 1.8 ± 0.8; VCI-ND, 2.7 ± 1.4; controls, 1.4 ± 0.6; *p* = 0.0033) was significantly larger in the VCI-ND patients than in the other two groups, suggesting an increase of the ICF.

### 3.2. Follow-Up

**Figure 2:**
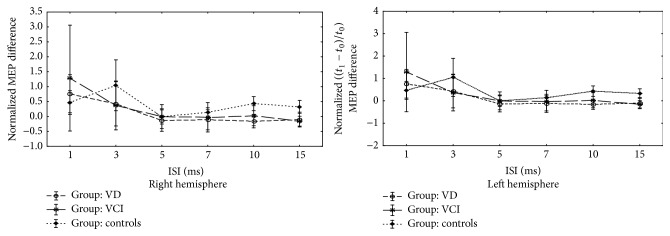
Comparison of the time course of intracortical excitability of patients and controls between baseline (*t*_0_) and follow-up (*t*_1_). Normalized MEP difference is computed as (*t*_1_ − *t*_0_)/*t*_0_. The comparison over time of the paired-pulse TMS curves between patients and controls did not show significant differences in terms of intracortical inhibition and intracortical facilitation between the three groups.* y*-axis shows the normalized MEP difference at baseline (*t*_0_) and at follow-up (*t*_1_) (computed as the value at time *t*_1_ minus the one at time *t*_0_ divided by *t*_0_). MEP, motor evoked potential; ISI, interstimulus interval; VD, patients with vascular depression; VCI, patients with vascular cognitive impairment-no dementia.

The comparison of neuropsychological and TMS characteristics of the three groups between baseline and follow-up is summarized in Tables 3 and 4, respectively. Unlike VCI-ND patients, depressed patients showed a significant decline of their functional status (IADL), together with worsening of the mean FAB score (Table 3). The TMS evaluation showed that the median rMT decreased significantly in both VCI-ND and VD patients (without significant difference between the two groups) compared to controls, whereas the CSP lengthened its duration from both hemispheres in controls but not in patients (Table 4).  Figure 2 shows the comparison over time of the paired-pulse TMS curves between patients and controls; in particular, the ICF of VCI-ND group becomes similar to that found in the other two groups.

Finally, the correlation between psychopathological and TMS variables revealed a positive correlation in VCI-ND group between ISI of 15 ms from left hemisphere at baseline and MMSE score at follow-up (rho = 0.604961; *p* < 0.0025; *p* value lowered according to Bonferroni correction). The correlation resulted to be significant even after controlling for the false discovery rate with Benjamini-Hochberg procedure (critical value set using 0.20 as false discovery rate).

### 3.3. Discussion

This is the first longitudinal study assessing the neurophysiology of late-onset depression as a potential risk factor for future VaD in patients with subcortical vascular disease. The high level of intracortical facilitation observed at baseline in nondepressed patients only might be protective from cognitive decline, possibly through an enhancement in glutamate-related neuroplasticity. Moreover, the hyperexcitability at single-pulse TMS observed at follow-up in both group of patients also points out involvement of glutamatergic neurotransmission, although without a specific neurophysiological change that parallels cognitive decline in depressed patients. This suggests that the mechanisms that contribute to cognitive deterioration in VD might be related either to subcortical changes produced by vascular lesions or to the lack of compensatory functional cortical.

These results are in line with previous TMS studies in subjects with subcortical vascular disease and clinical-cognitive features of VCI-ND, showing an enhanced ICF [40]. An increase of cortical excitability, together with significant worsening of frontal lobe abilities but without the development of dementia, was found after two years of follow-up [41]. Interestingly, a slight enhancement of ICF was also observed in patients with VD but not in those with early-onset major depression disorder [42, 43].

In the present study, monitoring vascular depressed and nondepressed individuals, the cortical excitability at follow-up increased significantly in both groups, although VD only showed clinical progression. This different behaviour may lie on the fact that the burden of subcortical vascular lesions constitutes a neuropathological platform for both cognitive decline and affective disorder in old age [7, 11]. In this context, the neurophysiological contribution might shed lights on the mechanisms underlying progression or preservation of cognitive functions in depression. In particular, an increased ICF, probably trough plastic compensatory phenomena involving the excitatory glutamatergic interneurons within the motor cortex, might act preserving cognition in VCI-ND [40, 41, 43]. Conversely, a lack of this hyperfacilitation in VD patients might contribute to their cognitive and functional deterioration, suggesting an impaired level of plasticity. This hypothesis is in agreement with a growing body of evidences indicating that the glutamate neurotransmission, which is known to play a major role in synaptic plasticity, is disrupted in depressive disorder and that drugs targeting the N-methyl-D-aspartate receptor have shown antidepressant properties [44].

It is noteworthy that the increased cortical excitability observed at follow-up might be also related to parallel degeneration of inhibitory GABAergic terminals. Indeed, in patients, we did not find the same prolongation of the CSP observed in the control group. Given that the CSP is a well-known measure of motor cortex inhibition largely mediated by GABA-B receptors [45], this finding might suggest a physiological role of the GABAergic transmission in controls with normal aging [45]; however, it should be kept in mind that discrepant results, probably related to technical and experimental differences, have emerged even in healthy adults [46–51].

Finally, in the last decade, research has been focused on the intriguing role of the neurotrophin release in mood disorders. The “neurogenic and neurotrophic hypothesis” assumes that development of depression would be, at least partially, related to the reduced neurogenesis and/or depletion of neurotrophic factors, which can eventually lead to functional impairment of brain network implicated in mood-affect regulation. In particular, serum level of brain-derived neurotrophic factor (BDNF) was found to be lower in late-onset depressed subjects than in age-matched controls [52, 53]. Low concentrations of BNDF and vascular endothelial growth factor (VEGF) can contribute to the progression of depression as well [54–56]. Other investigations have also addressed the relevance of the BDNF* Val66Met* polymorphism in depression; for instance, the* met* allele is associated with the incidence of poststroke depression [57] or with greater WMLs load in the elderly [58].

The main limitation of this study, as usual in TMS research, is the relatively small number of patients, although they were very homogeneous with a well-defined vascular risk profile. Secondly, although some TMS parameters change consistently with the involvement of different pathophysiological substrates even in the earliest stages of the disease [17], there is not pathognomonic measure, and therefore it cannot be excluded that, at this stage, TMS is not entirely able to quantify the risk of progression in patients with or without depression.

## 4. Conclusions

In conclusion, studying cortical excitability by means of TMS provides a potentially new window into the neurophysiological mechanisms behind depression of the late life and its reciprocal relationship with vascular-related cognitive disorders. TMS might also be used as the basis for a better understanding of the course of geriatric depression and for the development of therapeutic protocols based on nonpharmacological approach. Further independent investigations with larger group size are needed to confirm the present findings and to understand their modifications and clinical correlates over time.

## Figures and Tables

**(a):** 

	VD	VCI	Controls	Kruskal-Wallis ANOVA	
				*H*(2,42)	*p*
Age (years)	68.1 ± 8.6	70.0 ± 7.0	63.8 ± 7.2	4.04	0.132
Education (years)	7.5 ± 5.1	6.8 ± 4.0	9.7 ± 4.4	5.11	0.078
MMSE	26.6 ± 1.8	27.2 ± 2.1	28.5 ± 1.6	8.88	***0.0118***
ADL	5.8 ± 0.4	5.9 ± 0.3	6.0 ± 0.0	3.08	0.214
IADL	7.4 ± 1.4	7.8 ± 0.4	7.8 ± 0.4	0.39	0.823
HDRS	14.8 ± 6.1	4.2 ± 2.0	4.1 ± 2.3	29.28	***0***
AS	1.3 ± 0.5	0.4 ± 0.3	0.3 ± 0.4	23.68	***0***
SCID	1.9 ± 0.3	0.0 ± 0.0	0.0 ± 0.0	39.97	***0***
Stroop T	42.3 ± 15.5	43.9 ± 17.8	24.6 ± 12.5	12.80	***0.0017***
Stroop E	2.4 ± 2.5	3.8 ± 3.5	0.5 ± 0.6	12.94	***0.0015***
FAB	14.8 ± 2.2	14.3 ± 2.4	17.1 ± 1.5	15.35	***0.0005***

**(b):** 

*Post hoc* analysis	VD-VCI		VD-controls		VCI-controls	
	*Z*	*p*	*Z*	*p*	*Z*	*p*
MMSE	0.91	1.000	2.93	***0.0099***	1.75	0.240
HDRS	4.39	***0.00003***	4.74	***0.000006***	0.03	1
AS	3.52	***0.0013***	4.55	***0.00001***	0.64	1
SCID	4.37	***0.00004***	4.76	***0.000006***	0.00	1
Stroop T	0.24	1.000	3.10	***0.0059***	3.04	***0.0071***
Stroop E	0.86	1.000	2.67	***0.0224***	3.27	***0.0031***
FAB	0.45	1.000	3.28	***0.0031***	3.41	***0.0019***

VD, patients with vascular depression; VCI, patients with vascular cognitive impairment-no dementia; MMSE, mini mental state examination; ADL, Activity of Daily Living; IADL, Instrumental Activity of Daily Living; HDRS, 17-item Hamilton Depression Rating Scale; AS, Apathy Scale; Stroop T, Stroop color-word test interference-time (sec); Stroop E, Stroop color-word test interference-number of errors; FAB, frontal assessment battery; numbers in bold and italic font indicate statistically significant *p* value.

**Table 2:** Single-pulse TMS parameters obtained from patients and controls at baseline.

	VD	VCI	Controls	Kruskal-Wallis ANOVA	
				*H*(2,42)	*p*
*Left hemisphere*					
rMT (%)	47.2 ± 9.8	47.4 ± 9.0	43.0 ± 5.5	2.86	0.238
CSP (ms)	86.4 ± 38.3	93.2 ± 37.0	71.0 ± 16.6	3.22	0.200
MEP latency (ms)	19.6 ± 1.8	20.4 ± 1.3	20.3 ± 1.6	1.64	0.439
CMCT (ms)	5.7 ± 1.0	5.3 ± 0.4	6.0 ± 1.0	4.81	0.090
CMCT-F (ms)	5.3 ± 0.9	5.4 ± 0.8	5.9 ± 0.8	4.20	0.122
A ratio	0.4 ± 0.1	0.4 ± 0.1	0.3 ± 0.1	1.91	0.384
F amplitude (*μ*V)	0.1 ± 0.1	0.1 ± 0.0	0.1 ± 0.1	1.96	0.375

*Right hemisphere*					
rMT (%)	46.1 ± 9.6	44.1 ± 6.2	42.9 ± 4.6	1.54	0.463
CSP (ms)	97.1 ± 48.3	91.5 ± 36.8	67.7 ± 22.8	3.52	0.171
MEP latency (ms)	19.7 ± 1.7	20.1 ± 1.6	19.9 ± 1.6	0.62	0.732
CMCT (ms)	5.7 ± 0.8	5.5 ± 0.4	5.7 ± 1.0	0.13	0.936
CMCT-F (ms)	5.3 ± 0.7	5.5 ± 1.0	5.6 ± 1.1	0.51	0.775
A ratio	0.5 ± 0.2	0.4 ± 0.1	0.4 ± 0.1	1.24	0.538
F amplitude (*μ*V)	0.1 ± 0.1	0.2 ± 0.1	0.1 ± 0.1	3.98	0.137

VD, patients with vascular depression; VCI, patients with vascular cognitive impairment-no dementia; rMT, resting motor threshold; CSP, cortical silent period; MEP, motor evoked potential; CMCT, central motor conduction time; CMCT-F, central motor conduction time estimated with the F wave latency; A ratio, CMAP/MEP amplitude ratio.

**(a):** 

	VD		VCI		Controls		Kruskal-Wallis ANOVA	
	Mean	SD	Mean	SD	Mean	SD	*H*(2,40)	*p*
MMSE	−2.86	3.54	−1.00	1.73	−1.42	1.70	1.59	0.451
ADL	−0.67	1.35	−0.10	0.57	0.00	0.00	3.11	0.211
IADL	−1.20	1.52	−0.70	0.82	0.07	0.26	11.81	***0.0027***
HDRS	1.80	6.79	0.30	4.40	0.53	2.59	0.95	0.622
AS	−0.30	0.64	−0.08	0.34	−0.04	0.47	2.67	0.263
SCID	−0.07	0.46	0.20	0.63	—	—	1.21	0.545
Stroop T	10.47	25.18	16.03	31.14	5.43	14.18	1.03	0.597
Stroop E	1.47	3.45	−0.24	2.68	0.37	0.85	0.73	0.695
FAB	0.48	3.49	0.39	1.88	−1.14	1.35	8.45	***0.0146***

**(b):** 

*Post hoc* analysis	VD-VCI		VD-controls		VCI-controls	
	*Z*	*p*	*Z*	*p*	*Z*	*p*
IADL	0.39	1.000	2.76	***0.0171***	2.07	0.114
FAB	0.80	1.000	2.85	***0.0127***	1.75	0.239

VD, patients with vascular depression; VCI, patients with vascular cognitive impairment-no dementia; SD, standard deviation; MMSE, mini mental state examination; ADL, Activity of Daily Living; IADL, Instrumental Activity of Daily Living; HDRS, 17-item Hamilton Depression Rating Scale; AS, Apathy Scale; SCID, structured clinical interview for DSM-IV; Stroop T, Stroop color-word test interference-time (sec); Stroop E, Stroop color-word test interference-number of errors; FAB, frontal assessment battery; numbers in bold and italic font indicate statistically significant *p* value.

**(a):** 

	Wilcoxon matched-pairs test					
	VD		VCI-ND		Controls	
	*Z*	*p*	*Z*	*p*	*Z*	*p*
*Left hemisphere*						
rMT (%)	2.67	***0.0076***	2.66	***0.0076***	0.19	0.842
CSP (ms)	0.28	0.776	0.56	0.575	2.92	***0.0035***

*Right hemisphere*						
rMT (%)	2.10	***0.0353***	2.49	***0.0125***	0.90	0.367
CSP (ms)	1.41	0.158	0.82	0.407	2.86	***0.0042***

**(b):** 

	VD		VCI-ND		Controls		Kruskal-Wallis ANOVA	
	Mean	SD	Mean	SD	Mean	SD	*H*(2,40)	*p*
*Left hemisphere*								
rMT (%)	−5.27	6.53	−5.70	3.83	0.53	6.86	10.11	***0.0064***
CSP (ms)	1.10	25.92	3.20	24.26	32.61	27.73	6.62	***0.0365***
MEP latency (ms)	0.32	1.32	−0.09	0.54	0.41	1.48	1.77	0.412
CMCT (ms)	0.12	1.42	0.32	0.61	0.63	0.84	1.20	0.549
CMCT-F (ms)	−0.30	1.03	0.27	0.67	0.67	1.10	1.56	0.458
A ratio	0.03	0.19	−0.07	0.20	−0.05	0.07	3.84	0.146
F amplitude (*μ*V)	0.02	0.08	−0.04	0.07	−0.03	0.06	4.13	0.127

*Right hemisphere*								
rMT (%)	−3.93	5.83	−3.10	2.92	1.07	6.11	7.62	***0.0221***
CSP (ms)	15.83	41.03	4.20	15.18	31.21	33.85	3.53	0.171
MEP latency (ms)	0.11	0.81	0.15	1.17	−0.13	1.45	0.74	0.689
CMCT (ms)	−0.17	1.31	−0.05	0.79	−0.01	0.99	0.29	0.862
CMCT-F (ms)	−0.19	1.14	−0.46	2.20	0.01	1.05	0.41	0.813
A ratio	−0.02	0.27	−0.05	0.10	0.02	0.12	2.86	0.239
F amplitude (*μ*V)	0.01	0.07	−0.10	0.14	0.01	0.04	6.56	***0.0375***

**(c):** 

*Post hoc* analysis	VD-VCI		VD-controls		VCI-controls	
	*Z*	*p*	*Z*	*p*	*Z*	*p*
rMT (left)	0.75	1.000	2.41	***0.0474***	2.90	***0.0108***
CSP (left)	0.09	1.000	2.34	0.0574	1.99	0.137
rMT (right)	0.37	1.000	2.15	0.0934	2.30	0.064
F amplitude (right)	2.30	0.062	0.02	1.000	2.28	0.066

VD, patients with vascular depression; VCI, patients with vascular cognitive impairment-no dementia; SD, standard deviation; rMT, resting motor threshold; CSP, cortical silent period; ISI, interstimulus interval; numbers in bold and italic font indicate statistically significant *p* value.
